# Notes and Notices

**Published:** 1894-03

**Authors:** 


### NOTES AND NOTICES.

THE MISSOURI INSTITUTE OF HOMŒOPATHY will hold its Eighteenth Annual Session in St. Louis, on Tuesday, Wednesday, and Thursday, April 17th, 18th, and 19th. The Missouri Institute is so well known for the excellency of its meetings that it needs no eulogies. An unusually good corps of chairmen have secured an exceptionally good list of papers from distinguished physicians, and the success of the meeting is thereby assured. Complete announcement and programme in April. Your presence as a participant is very much desired. H. J. RAVOLD, M. D., *General Secretary.*

E. B. TREAT, publisher, has in press the following books, which will be issued early in 1894:

A MANUAL OF CLINICAL DIAGNOSIS, by Albert Abrams, M. D., Assistant Professor Clinical Medicine and Demonstrator of Pathology, Cooper Medical College, San Francisco, new and enlarged edition, $2.75.

DISEASES OF THE HAIR AND SCALP, by Geo. T. Jackson, M. D., Chief of Dermatological Clinic, College of Physicians and Surgeons, New York, illustrated, second edition, enlarged, $2.75.

HOW TO USE THE FORCEPS: A Manual of the Obstretric Art and Mechanism of Labor, by Prof. H. G. Landis, M. D., Columbus, O., enlarged edition; by C. H. Bushong, M. D., New York, $1.75.

Also just issued: PRACTICAL HYGIENE, based on modern theories and scientific progress, by C. G. Currier, M. D., New York, Specialist and Expert in Sanitary Science, $2.75.

CO-PARTNERSHIP NOTICE.—On and after January 1st, 1894, J. M. Selfridge M. D., and C. M. Selfridge, M. D., will be co-partners in the practice of medicine and surgery. All persons indebted to the undersigned are requested to call and settle.

Oakland, December 27th, 1893.

J. M. SELFRIDGE, M. D.,
1068 Broadway, Oakland, California.

